# Psoriasis as a Potential Risk Factor for Inflammatory Bowel Disease: Findings from a Nationally Representative Korean Population

**DOI:** 10.3390/biomedicines13102334

**Published:** 2025-09-24

**Authors:** Ho Suk Kang, Kyeong Min Han, Joo-Hee Kim, Dae Myoung Yoo, Hyo Geun Choi, Nan Young Kim, Kyueng-Whan Min, Mi Jung Kwon

**Affiliations:** 1 Division of Gastroenterology, Department of Internal Medicine, Hallym University Sacred Heart Hospital, Hallym University College of Medicine, Anyang 14068, Republic of Korea; putamenn@hanmail.net; 2 Hallym Data Science Laboratory, Hallym University College of Medicine, Anyang 14068, Republic of Korea; kmhan@hallym.ac.kr (K.M.H.); ydm@hallym.ac.kr (D.M.Y.); 3 Division of Pulmonary, Allergy and Critical Care Medicine, Department of Internal Medicine, Hallym University Sacred Heart Hospital, Hallym University College of Medicine, Anyang 14068, Republic of Korea; luxjhee@hallym.or.kr; 4 Suseo Seoul E.N.T. Clinic, 10, Bamgogae-ro 1-gil, Gangnam-gu, Seoul 06349, Republic of Korea; mdanalytics@naver.com; 5 Hallym Institute of Translational Genomics and Bioinformatics, Hallym University Medical Center, Anyang 14068, Republic of Korea; honeyny@hallym.or.kr; 6 Department of Pathology, Uijeongbu Eulji Medical Center, Eulji University School of Medicine, 712, Dongil-ro, Uijeongbu 11759, Republic of Korea; kyueng@gmail.com; 7 Department of Pathology, Hallym University Sacred Heart Hospital, Hallym University College of Medicine, Anyang 14068, Republic of Korea

**Keywords:** psoriasis, inflammatory bowel diseases, Crohn’s disease, ulcerative colitis, nested case–control study

## Abstract

**Background/Objectives**: Psoriasis is a chronic immune-mediated disorder that may share pathogenic pathways with inflammatory bowel disease (IBD), including ulcerative colitis (UC) and Crohn’s disease (CD). Although Western studies suggest a possible link between psoriasis and increased IBD risk, large-scale evidence from Asian populations remains limited and inconsistent. Using nationwide Korean cohort data, we aimed to investigate this association. **Methods**: In this retrospective, nested case–control study using the Korean National Health Insurance Service–National Sample Cohort (2002–2019), incident IBD cases were matched 1:4 to controls by age, sex, income, region, and index date. Psoriasis history prior to the IBD index date was identified using diagnostic codes. Overlap propensity score weighting balanced covariates, and weighted multivariable logistic regression estimated adjusted odds ratios (aORs) with 95% confidence intervals (CIs). **Results**: Among 10,966 IBD patients and 43,864 controls, psoriasis was associated with higher IBD risk (aOR 1.63; 95% CI, 1.38–1.93). The association was stronger for UC (aOR 1.77; 95% CI, 1.44–2.18) than for CD (aOR 1.37; 95% CI, 1.01–1.84). UC risk was elevated across most subgroups, whereas CD risk reached significance only in individuals < 45 years. **Conclusions**: In this large, nationally representative Korean cohort, psoriasis was linked to an increased risk of subsequent IBD, particularly UC. Although causality cannot be inferred, these findings may underscore the importance of considering possible gastrointestinal comorbidity in patients with psoriasis.

## 1. Introduction

Psoriasis is a chronic, systemic, immune-mediated condition characterized by recurrent erythematous plaques [1]. Its prevalence ranges from approximately 0.14% in East Asia to 1.92% in Western Europe, with estimates of 0.34% in Japan, 0.59% in China, and 0.44–0.45% in Korea [1,2,3,4]. Beyond cutaneous manifestations, psoriasis is increasingly recognized as a systemic disease, with up to 30% of patients developing psoriatic arthritis and many experiencing comorbidities such as cardiovascular disease, chronic kidney disease, metabolic syndrome, and psychiatric disorders [5,6]. These systemic associations contribute to reduced quality of life, psychiatric burden, and increased healthcare costs [7]. 

Psoriasis is part of the broader spectrum of immune-mediated inflammatory dis-eases, in which the presence of one condition increases susceptibility to others. Inflammatory bowel disease (IBD)—encompassing ulcerative colitis (UC) and Crohn’s dis-ease (CD)—is a representative example, marked by chronic, relapsing gastrointestinal inflammation that requires long-term management [8,9]. Although clinically distinct, psoriasis and IBD share core pathogenic mechanisms, including immune dysregulation, Th17-mediated inflammation, and overlapping susceptibility loci such as IL23R and IL12B [10,11,12]. Large registry-based studies from Western countries, including Denmark and Sweden, have consistently demonstrated 1.5–2.0-fold higher risks of UC and CD in patients with psoriasis [10,13]. These findings are further supported by the concept of the gut–skin axis, whereby dysbiosis and impaired barrier function promote systemic immune activation [14,15,16].

Historically considered a Western disease, IBD incidence has risen sharply in Asia, including in Korea [8,9,17,18,19,20]. The current Korean prevalence is 11.24 per 100,000 for CD and 30.87 for UC, with both showing marked increases over the past three decades [9,17,18,21]. Nationwide insurance data indicate a three- to fivefold rise in annual incidence, with the steepest increase among adults aged 20–40 years—the peak of working age—highlighting substantial socioeconomic and quality-of-life consequences [22,23,24]. Recent shifts in environmental exposures—such as rapid industrialization, urbanization, increased antibiotic use, and Westernized dietary patterns with reduced fiber intake—are thought to impair epithelial barrier function and alter gut microbiota, potentially influencing the occurrence of both conditions, particularly in Asia [8]. Although IBD incidence has risen sharply in Asia, including in Korea [8,9,17,18,19,20], prior Asian studies were often constrained by small sample sizes, heterogeneous designs, and incomplete adjustment for confounders [25,26]. Meta-analyses that include Asian cohorts are largely dominated by Western data, leaving uncertainty regarding the impact of population-specific genetics, environmental exposures, and regional healthcare factors [13,27]. Moreover, the relatively lower prevalence of CD in East Asia may further reduce statistical power to detect associations [26,28].

Given these gaps, large-scale population-based studies in Asian settings are essential to clarify whether psoriasis is independently associated with IBD risk. Using a nationally representative Korean cohort, we investigated this association and examined whether the magnitude and pattern of risk differ by IBD subtype and across clinically relevant subgroups.

## 2. Materials and Methods 

### 2.1. Ethics Statement and Data Source

This retrospective, nested case–control study was approved by the Institutional Review Board of Hallym University (Approval No: 2022-10-008) and conducted in accordance with the Declaration of Helsinki. The requirement for written informed consent was waived due to the use of pre-existing administrative data. The study adhered to national ethical guidelines for observational research and was reported according to the STROBE (Strengthening the Reporting of Observational Studies in Epidemiology) statement.

We utilized data from the Korean National Health Insurance Service–National Sample Cohort (KNHIS–NSC), which includes a 2.2% random sample of the Korean population in 2002, stratified by age (18 categories), sex, and income level (41 categories) [29]. Participants were followed from 2002 to 2019 unless lost due to death or emigration. The dataset contains longitudinal health information for 1,137,861 individuals and 219,673,817 medical claims.

### 2.2. Study Population and Case Definition

From the NHIS–NSC, we identified patients diagnosed with IBD, defined as Crohn’s disease (CD, ICD-10: K50) or ulcerative colitis (UC, ICD-10: K51). A total of 12,943 IBD cases were identified between 2002 and 2019. To exclude pre-existing cases, individuals diagnosed in 2002 were removed (1-year washout, *n* = 1977). 

The control population consisted of individuals without IBD during the study period (*n* = 1,124,918). Each IBD case was exactly matched (1:4) to controls by sex, age, income level, region, and index date (date of first IBD diagnosis for cases and corresponding date for matched controls). After matching, propensity scores were calculated using baseline covariates, and overlap propensity score weighting was applied to achieve covariate balance. Matching excluded 1,081,054 controls, resulting in 10,966 IBD cases and 43,864 matched controls for analysis.

### 2.3. Exposure Assessment

Psoriasis history prior to the index date was identified using the following ICD-10 codes: L40.0, L40.1, L40.2, L40.4, L40.5, L40.8, L40.9, M07.0, M07.1, M07.2, M07.3, and M09.0. To improve diagnostic accuracy, psoriasis was defined as ≥2 outpatient visits with a relevant code [30]. Psoriasis subtypes were identified and described for completeness; however, they were included only descriptively, and no differentiated analyses by subtype were conducted.

### 2.4. Final Cohort

**Figure 1:**
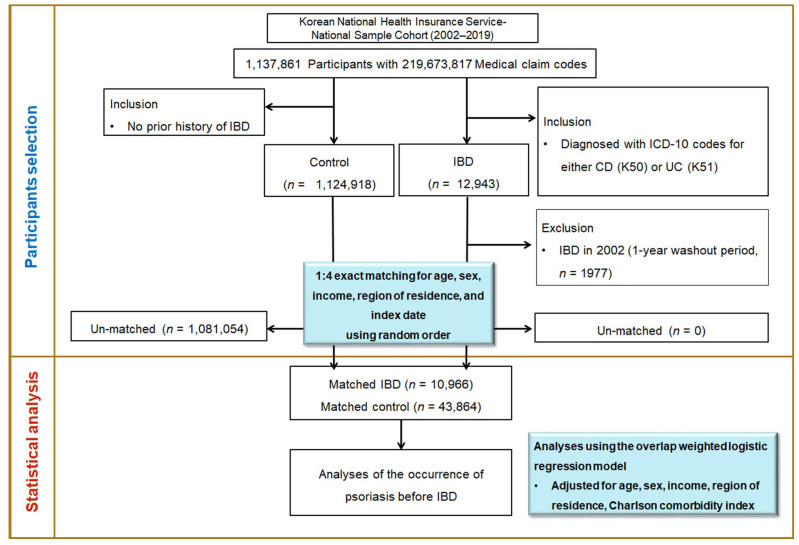
Flowchart of participant selection and study design. This study analyzed data from the Korean National Health Insurance Service–National Sample Cohort (2002–2019), which includes 1,137,861 individuals and over 219 million medical claims. Inflammatory bowel disease (IBD) cases were identified using ICD-10 codes (K50 for Crohn’s disease (CD) and K51 for ulcerative colitis (UC)). Controls were selected from individuals with no history of IBD. After exclusions, 1:4 exact matching was performed based on age, sex, income, region, and index date. The final sample included 10,966 IBD cases and 43,864 matched controls. And then, psoriasis history was assessed prior to IBD diagnosis.

From a total of 1,137,861 participants and 219,673,817 medical claim records collected, the final study cohort comprised 54,830 participants (10,966 IBD cases and 43,864 controls). Psoriasis history was tracked for all participants from cohort entry until the index date (Figure 1).

### 2.5. Covariates and Statistical Analysis

Participants were categorized into 18 age groups (5-year intervals), five income levels (1 = lowest to 5 = highest), and residential areas classified as urban or rural based on 16 administrative districts. In addition to exact matching on demographic and socioeconomic variables, comorbidity burden was assessed using the Charlson Comorbidity Index (CCI), which assigns scores from 0 to 29 based on 17 predefined comorbidities [31], and was included as a covariate in our overlap propensity score–weighted models to account for underlying health conditions.

To minimize baseline differences between IBD cases and controls, we applied propensity score overlap weighting. Propensity scores were estimated via multivariable logistic regression including all covariates. Overlap weights were calculated as 1–propensity score for IBD cases and propensity score for controls [19]. Covariate balance between groups was evaluated using standardized differences, which quantify the difference in means or proportions between groups relative to the pooled standard deviation. Unlike *p* values, standardized differences are not affected by sample size and provide a more reliable measure of balance in observational studies. An absolute standardized difference ≤ 0.20 was considered indicative of adequate balance across covariates.

We used multivariable logistic regression with overlap weighting to estimate crude and adjusted odds ratios (ORs) and 95% confidence intervals (CIs) for the association between psoriasis history and subsequent IBD development. Adjusted models included age, sex, income level, residential area, and CCI score. Subgroup analyses were conducted for all covariates to explore potential effect modification.

All statistical analyses were performed using SAS version 9.4 (SAS Institute, Cary, NC, USA). Two-sided *p* values < 0.05 were considered statistically significant.

## 3. Results

The baseline characteristics of 10,966 patients with IBD and 43,864 matched controls, matched for age, sex, income, and region of residence, are summarized in Table 1. Matching achieved excellent baseline comparability, with standardized differences of 0.00 for all matched variables. After applying overlap weighting to account for additional covariates, standardized differences for all variables were further reduced to 0, indicating near-perfect balance between the IBD and control groups.

As shown in Table 2, propensity score overlap-weighted multivariable logistic regression analysis revealed that psoriasis was significantly linked to an increased likelihood of developing IBD (crude OR = 1.63; 95% CI: 1.34–1.98; *p* < 0.001; adjusted OR = 1.63; 95% CI: 1.38–1.93; *p* < 0.001). When analyzed by disease type, psoriasis was related to significantly higher odds of UC (adjusted OR = 1.77; 95% CI: 1.44–2.18; *p* < 0.001) and a modest but statistically significant increase in CD likelihood (adjusted OR = 1.37; 95% CI: 1.01–1.84; *p* = 0.041).

**Figure 2:**
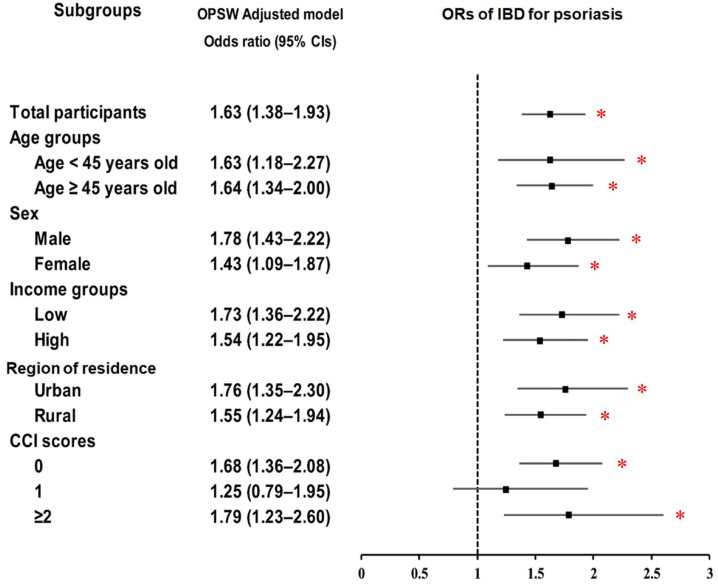
Forest plot of odds ratios (ORs) (95% confidence intervals (CI)) for the association between psoriasis history and the subsequent occurrence of inflammatory bowel diseases (IBD), including subgroup analyses by age, sex, income, residential area, and Charlson comorbidity index (CCI). Abbreviations: OPSW; overlap propensity score weighting. * Significance at *p* < 0.05.

Subgroup analyses (Figure 2; Table S1) demonstrated consistent positive associations between psoriasis history and IBD risk across demographic and clinical strata, including age, sex, income, residential area, and CCI score. The association was particularly pronounced among individuals with lower income (OR = 1.73; 95% CI: 1.36–2.22; *p* < 0.001), those residing in urban areas (OR = 1.76; 95% CI: 1.35–2.30; *p* < 0.001), and those with higher comorbidity burden (CCI ≥ 2; OR = 1.79; 95% CI: 1.23–2.60; *p* = 0.002).

**Figure 3:**
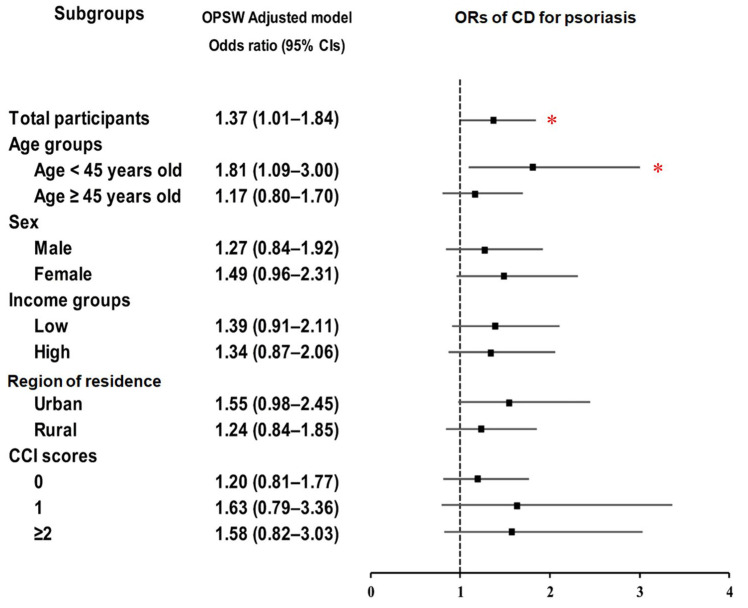
Forest plots of (ORs) (95% confidence intervals (CI)) for the association between psoriasis history and the subsequent occurrence of Crohn’s disease (CD), including subgroup analyses by age, sex, income, residential area, and Charlson comorbidity index (CCI). Abbreviations: OPSW; overlap propensity score weighting. * Significance at *p* < 0.05.

**Figure 4:**
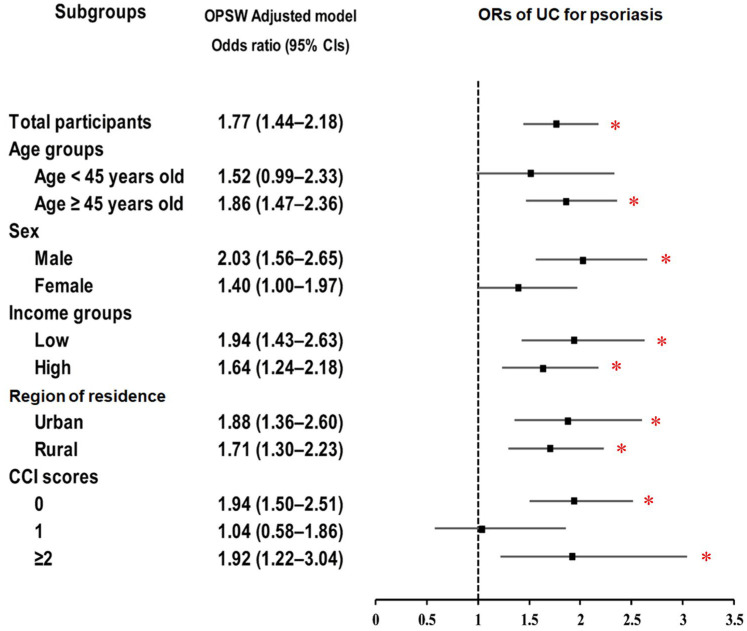
Forest plots of (ORs) (95% confidence intervals (CI)) for the association between psoriasis history and the subsequent occurrence of ulcerative colitis (UC), including subgroup analyses by age, sex, income, residential area, and Charlson comorbidity index (CCI). Abbreviations: OPSW; overlap propensity score weighting. * Significance at *p* < 0.05.

For CD, psoriasis showed an increased risk, reaching statistical significance only in certain subgroups, notably individuals <45 years (OR = 1.81; 95% CI: 1.09–3.00; *p* = 0.021) (Figure 3; Table S2). In contrast, psoriasis showed a consistent association with UC across most subgroups, with stronger effects in males (OR = 2.03; 95% CI: 1.56–2.65; *p* < 0.001), low-income individuals (OR = 1.94; 95% CI: 1.43–2.63; *p* < 0.001), and those with CCI ≥ 2 (OR = 1.92; 95% CI: 1.22–3.04; *p* = 0.005) (Figure 4; Table S3).

## 4. Discussion

In this large, nationwide Korean cohort study, a history of psoriasis was associated with a 63% increased risk of developing IBD, including both UC (adjusted OR = 1.77) and CD (adjusted OR = 1.37), after adjustment for sociodemographic and clinical factors. The association was stronger and more consistent for UC, whereas the link with CD reached statistical significance only among individuals younger than 45 years. Although the observed effect sizes were modest, they may still hold clinical significance: given the chronic nature and substantial morbidity of both psoriasis and IBD, even a 30–70% relative increase in risk could represent considerable burden at the population level [26,27]. The stronger associations observed for UC—particularly among men, younger individuals, and those with higher comorbidity burden—may help inform risk stratification and raise clinical awareness. These findings underscore the importance of vigilance for gastrointestinal symptoms in patients with psoriasis and support timely referral and management.

Our results are broadly consistent with large-scale studies from Western populations, which have generally reported elevated risks for both UC and CD among psoriasis patients [13,27]. For example, registry-based cohort studies from Denmark and Sweden, each including more than 20,000 patients, demonstrated hazard ratios between 1.5 and 2.0 for both subtypes, persisting after long-term follow-up [10]. Similarly, a U.S. administrative claims analysis found higher IBD prevalence in psoriasis patients—1.4% for overall IBD, 1.6% for CD, and 1.3% for UC—suggesting notable gastrointestinal comorbidity [32]. A meta-analysis of primarily Western cohorts, encompassing more than 7.7 million participants, reported pooled ORs of 1.70 for CD and 1.75 for UC in case–control studies, with even stronger associations in cohort designs [27]. 

Several biological mechanisms plausibly underlie the observed association. Both psoriasis and IBD involve dysregulation of the Th17 immune pathway and overexpression of cytokines such as IL-17 and IL-22, which sustain chronic inflammation in the skin and gastrointestinal tract [11,12]. The gut–skin axis has also been proposed, describing bidirectional interactions between intestinal and cutaneous immune responses [14]. The skin and gut share key features, including abundant microbial diversity and a rich blood supply, and their epithelial immune responses are strongly influenced by microbiota-derived metabolites [14,16,33]. Dysbiosis in either site—characterized by reduced microbial diversity and depletion of beneficial taxa such as *Akkermansia*, *Ruminococcus*, and *Faecalibacterium prausnitzii*—has been reported in both conditions, alongside enrichment of pro-inflammatory pathobionts [15,16,34]. These microbial shifts can impair barrier integrity, enhance systemic immune activation, and perpetuate multi-organ inflammation [14,15,16].

In contrast, Asian evidence remains scarce, limited to four published epidemiologic studies, and findings have been less consistent [25,26,28,35]. A previous Korean study reported higher risks of both CD (hazard ratio 1.95; 95% CI, 1.42–2.67) and UC (hazard ratio 1.65; 95% CI, 1.39–1.96) in patients with psoriatic disease, but it examined a broader autoimmune disease spectrum rather than IBD specifically [35]. A population-based study from Taiwan involving more than 2 million individuals reported a significant relevance between psoriasis and UC (hazard ratio = 1.49; 95% CI: 1.18–1.87) but no significant link with CD [26]. However, as the study primarily aimed to characterize the epidemiology and multiple comorbidities of psoriasis rather than IBD specifically, and because the number of IBD cases was relatively small, the findings should be interpreted with caution [26]. Similarly, a Japanese claims database analysis noted a trend toward increased UC risk without a statistically significant CD association; among 681 psoriasis patients, 1.2% had UC and 0.3% had CD, with variable diagnostic sequences between the two diseases. These results may partly reflect the lower baseline incidence of CD in East Asia or potential bias [28]. 

In our study, psoriasis was consistently associated with UC across most subgroups, with stronger effects observed among men, low-income individuals, and those with higher comorbidity burdens. By contrast, the CD association was confined to younger individuals (<45 years). Of note, the Japanese study also reported a male predominance among psoriasis patients with UC, although no age-related trend was observed, and comorbidity profiles were largely similar between groups [28]. Differences by subtype and region may relate to genetic architecture. A meta-analysis identified 11 susceptibility loci shared between psoriasis and IBD, but only four—IL23R, IL12B, REL, and TYK2—showed strong overlap for CD [36,37,38], suggesting weaker genetic linkage compared with UC. In addition, regional variation in healthcare access, diagnostic practices, environmental exposures (e.g., diet, microbiome composition, urbanization), and background IBD incidence may further contribute to heterogeneity in reported associations [13,26,28].

A key strength of our study is the use of KNHIS-NSC, a large, nationally representative dataset containing comprehensive longitudinal information on diagnoses, prescriptions, and sociodemographic factors [29,39]. We also applied robust design features, including 1:4 exact matching on key sociodemographic variables followed by overlap propensity score weighting, achieving excellent covariate balance (absolute standardized differences ≤ 0.2) for all critical variables, including comorbidity burden [40]. This approach yielded well-matched study groups (10,966 IBD patients and 43,864 controls) and improved causal inference by approximating a randomized controlled trial framework [41].

However, several limitations should be acknowledged. First, the retrospective observational design precludes definitive causal inference. Second, reliance on administrative claims data may have introduced misclassification bias and limited the availability of key variables. Our classification of IBD into UC and CD was based on administrative diagnostic codes, which follow the traditional dichotomous framework [35]. However, the recent literature suggests that IBD subtypes may exist along a more complex and overlapping spectrum, and reliance on historical coding may not fully reflect this nuance [42]. This limitation should be considered when interpreting our results. Although we adjusted for multiple sociodemographic and comorbidity factors, important risk determinants such as smoking status, alcohol consumption, dietary patterns, body mass index, and family history were unavailable. In addition, clinical information on psoriasis and IBD—including disease severity, duration, lesion extent, severity indices, and systemic treatment use—was not accessible. The absence of these data restricted our ability to evaluate potential effect modification and may have introduced residual confounding, partly explaining differences compared with other cohorts. Furthermore, as our study was restricted to the Korean population, the generalizability of the findings may be limited. Differences in genetic background, environmental exposures, and healthcare systems are important considerations when extrapolating our results: genetic susceptibility loci may vary across populations, environmental factors such as diet, microbiome composition, smoking prevalence, and antibiotic use differ regionally, and variations in healthcare access, diagnostic practices, and coding accuracy may all influence disease patterns [1,2,14,38]. These contextual factors should be carefully considered when applying our findings beyond Korea. Finally, no formal adjustment for multiple testing was applied in the subgroup analyses; thus, these results should be regarded as exploratory and interpreted with caution. Nonetheless, the consistency of the associations—particularly for UC—supports the robustness of our main findings. Future investigations incorporating detailed lifestyle and clinical information are warranted to further clarify these associations.

## 5. Conclusions

In summary, psoriasis was associated with modestly increased risks of both UC and CD in this large, nationally representative Korean cohort, with the UC association appearing more consistent across subgroups. Despite the modest effect sizes, their consistency suggests potential clinical relevance, particularly for UC, underscoring the importance of heightened vigilance for gastrointestinal symptoms in patients with psoriasis. Subgroups with higher observed UC risk—such as men, individuals with lower income, and those with greater comorbidity burden—may warrant closer evaluation. While these findings strengthen the epidemiologic evidence for a psoriasis–IBD link, they should be interpreted with caution given the observational design and potential residual confounding. 

## Figures and Tables

**Table 1:** General characteristics of participants.

Characteristics	Before Overlap PS Weighting Adjustment			After Overlap PS Weighting Adjustment		
	IBD	Control	Standardized Difference	IBD	Control	Standardized Difference
Age (n, %)			0.00			0.00
0–4	355 (3.24)	1420 (3.24)		284 (3.24)	284 (3.24)	
5–9	306 (2.79)	1224 (2.79)		245 (2.79)	245 (2.79)	
10–14	361 (3.29)	1444 (3.29)		289 (3.30)	289 (3.30)	
15–19	551 (5.02)	2204 (5.02)		440 (5.03)	440 (5.03)	
20–24	655 (5.97)	2620 (5.97)		524 (5.98)	524 (5.98)	
25–29	734 (6.69)	2936 (6.69)		587 (6.70)	587 (6.70)	
30–34	806 (7.35)	3224 (7.35)		644 (7.36)	644 (7.36)	
35–39	769 (7.01)	3076 (7.01)		614 (7.01)	614 (7.01)	
40–44	901 (8.22)	3604 (8.22)		720 (8.22)	720 (8.22)	
45–49	925 (8.44)	3700 (8.44)		739 (8.44)	739 (8.44)	
50–54	994 (9.06)	3976 (9.06)		796 (9.08)	796 (9.08)	
55–59	884 (8.06)	3536 (8.06)		705 (8.05)	705 (8.05)	
60–64	770 (7.02)	3080 (7.02)		614 (7.01)	614 (7.01)	
65–69	765 (6.98)	3060 (6.98)		610 (6.97)	610 (6.97)	
70–74	572 (5.22)	2288 (5.22)		455 (5.20)	455 (5.20)	
75–79	348 (3.17)	1392 (3.17)		277 (3.17)	277 (3.17)	
80–84	184 (1.68)	736 (1.68)		147 (1.67)	147 (1.67)	
85+	86 (0.78)	344 (0.78)		68 (0.78)	68 (0.78)	
Sex (n, %)			0.00			0.00
Male	5747 (52.41)	22,988 (52.41)		4590 (52.41)	4590 (52.41)	
Female	5219 (47.59)	20,876 (47.59)		4167 (47.59)	4167 (47.59)	
Income (n, %)			0.00			0.00
1 (lowest)	1833 (16.72)	7332 (16.72)		1462 (16.70)	1462 (16.70)	
2	1554 (14.17)	6216 (14.17)		1241 (14.17)	1241 (14.17)	
3	1992 (18.17)	7968 (18.17)		1590 (18.16)	1591 (18.16)	
4	2533 (23.10)	10,132 (23.10)		2024 (23.11)	2024 (23.11)	
5 (highest)	3054 (27.85)	12,216 (27.85)		2440 (27.86)	2440 (27.86)	
Region of residence (n, %)			0.00			0.00
Urban	4882 (44.52)	19,528 (44.52)		3901 (44.55)	3901 (44.55)	
Rural	6084 (55.48)	24,336 (55.48)		4856 (55.45)	4856 (55.45)	
CCI score (Mean, SD)	0.72 (1.48)	0.58 (1.35)	0.10	0.69 (1.28)	0.69 (0.68)	0.00
Psoriasis (n, %)	141 (1.29)	349 (0.80)	0.05	113 (1.28)	69 (0.79)	0.05

Abbreviations: CCI, Charlson Comorbidity Index; IBD, inflammatory bowel disease; PS, propensity score; SD, standard deviation.

**Table 2:** Crude and overlap propensity score weighted odds ratios of IBD, CD, and UC for psoriasis.

Characteristics	N of Event	N of Control	Odds Ratio (95% Confidence Interval)			
	(Exposure/Total, %)	(Exposure/Total, %)	Crude	*p*	Overlap PS Weighted Model †	*p*
Odds ratio of IBD (*n* = 54,830)						
Psoriasis	141/10,966 (1.3)	349/43,864 (0.8)	1.63 (1.34–1.98)	<0.001 *	1.63 (1.38–1.93)	<0.001 *
Control	10,825/10,966 (98.7)	43,515/43,864 (99.2)	1		1	
Odds ratio of CD (*n* = 23,185)						
Psoriasis	41/4637 (0.9)	120/18,548 (0.6)	1.37 (0.96–1.96)	0.083	1.37 (1.01–1.84)	0.041 *
Control	4596/4637 (99.1)	18,428/18,548 (99.4)	1		1	
Odds ratio of UC (*n* = 31,645)						
Psoriasis	100/6329 (1.6)	229/25,316 (0.9)	1.76 (1.39–2.23)	<0.001 *	1.77 (1.44–2.18)	<0.001 *
Control	6229/6329 (98.4)	25,087/25,316 (99.1)	1		1	

Abbreviations: IBD, inflammatory bowel disease; CD, Crohn’s disease; UC, ulcerative colitis; PS, propensity score; * Significance at *p* < 0.05. † Adjusted for age, sex, income, region of residence, and CCI scores.

## Data Availability

Restrictions apply to the availability of these data. The data were obtained from the Korean National Health Insurance Sharing Service (NHISS) (accessed on 1 March 2025) and are available at https://nhiss.nhis.or.kr.

## Supplementary Materials

The following supporting information can be downloaded at https://www.mdpi.com/article/10.3390/biomedicines13102334/s1, Table S1: Crude and overlap propensity score weighted odd ratios of psoriasis for IBD; Table S2: Crude and overlap propensity score weighted odd ratios of CD for psoriasis; Table S3: Crude and overlap propensity score weighted odd ratios of UC for psoriasis.
