# Supplementary material for: Psoriasis as a Potential Risk Factor for Inflammatory Bowel Disease: Findings from a Nationally Representative Korean Population
Source: Biomedicines. 2025 Sep 24;13(10):2334. doi: 10.3390/biomedicines13102334 (PMC12562003; doi:10.3390/biomedicines13102334)
Supplement: Supplementary file 1 [file biomedicines-13-02334-s001.zip › biomedicines-3856773-supplementary.pdf]

Table S1 Crude and overlap propensity score weighted odd ratios of psoriasis for IBD

Characteristics	N of IBD	N of Control	Odd ratios for IBD (95% confidence interval)			
	(exposure/total, %)	(exposure/total, %)	Crude	p value	Overlap weighted model †	p value
Total participants (n = 54,830)						
Psoriasis	141/10,966 (1.3)	349/43,864 (0.8)	1.63 (1.34–1.98)	<0.001*	1.63 (1.38–1.93)	<0.001*
Control	10,825/10,966 (98.7)	43,515/43,864 (99.2)	1		1	
Age < 45 years old (n = 27,190)						
Psoriasis	38/5438 (0.7)	94/21,752 (0.4)	1.62 (1.11–2.37)	0.012*	1.63 (1.18–2.27)	0.003*
Control	5400/5438 (99.3)	21,658/21,752 (99.6)	1		1	
Age ≥ 45 years old (n = 27,640)						
Psoriasis	103/5528 (1.9)	255/22,112 (1.2)	1.63 (1.29–2.05)	<0.001*	1.64 (1.34–2.00)	<0.001*
Control	5425/5528 (98.1)	21,857/22,112 (98.8)	1		1	
Male (n = 28,735)						
Psoriasis	89/5747 (1.5)	203/22,988 (0.9)	1.77 (1.37–2.27)	<0.001*	1.78 (1.43–2.22)	<0.001*
Control	5658/5747 (98.5)	22,785/22,988 (99.1)	1		1	
Female (n = 26,095)						
Psoriasis	52/5219 (1.0)	146/20,876 (0.7)	1.43 (1.04–1.96)	0.028*	1.43 (1.09–1.87)	0.009*
Control	5167/5219 (99.0)	20,730/20,876 (99.3)	1		1	
Low income group (n = 26,895)						
Psoriasis	70/5379 (1.3)	162/21,516 (0.8)	1.74 (1.31–2.31)	<0.001*	1.73 (1.36–2.22)	<0.001*
Control	5309/5379 (98.7)	21,354/21,516 (99.2)	1		1	
High income group (n = 27,935)						
Psoriasis	71/5587 (1.3)	187/22,348 (0.8)	1.53 (1.16–2.01)	0.003*	1.54 (1.22–1.95)	<0.001*
Control	5516/5587 (98.7)	22,161/22,348 (99.2)	1		1	
Urban resident (n = 24,410)						
Psoriasis	61/4882 (1.2)	139/19,528 (0.7)	1.77 (1.31–2.39)	<0.001*	1.76 (1.35–2.30)	<0.001*
Control	4821/4882 (98.8)	19,389/19,528 (99.3)	1		1	
Rural resident (n = 30,420)						
Psoriasis	80/6084 (1.3)	210/24,336 (0.9)	1.53 (1.18–1.98)	0.001*	1.55 (1.24–1.94)	<0.001*
Control	6004/6084 (98.7)	24,126/24,336 (99.1)	1		1	
CCI scores = 0 (n = 40,324)						
Psoriasis	85/7572 (1.1)	224/32,752 (0.7)	1.65 (1.28–2.12)	<0.001*	1.68 (1.36–2.08)	<0.001*
Control	7487/7572 (98.9)	32,528/32,752 (99.3)	1		1	
CCI scores = 1 (n = 6798)						
Psoriasis	20/1592 (1.3)	57/5206 (1.1)	1.15 (0.69–1.92)	0.595	1.25 (0.79–1.95)	0.338
Control	1572/1592 (98.7)	5149/5206 (98.9)	1		1	
CCI scores ≥ 2 (n = 7708)						
Psoriasis	36/1802 (2.0)	68/5906 (1.2)	1.75 (1.17–2.63)	0.007*	1.79 (1.23–2.60)	0.002*
Control	1766/1802 (98.0)	5838/5906 (98.8)	1		1	

Abbreviations: IBD, inflammatory bowel disease; CCI, Charlson Comorbidity Index.

* Significance at $p < 0.05$.

† Adjusted for age, sex, income, region of residence, and CCI scores.

Table S2 Crude and overlap propensity score weighted odd ratios of psoriasis for CD

Characteristics	N of CD (exposure/total, %)	N of Control (exposure/total, %)	Odd ratios for CD (95% confidence interval) Crude	p value	Overlap weighted model †	p value
Total participants (n = 23,185)						
Psoriasis	41/4637 (0.9)	120/18,548 (0.6)	1.37 (0.96–1.96)	0.083	1.37 (1.01–1.84)	0.041*
Control	4596/4637 (99.1)	18,428/18,548 (99.4)	1		1	
Age < 45 years old (n = 13,810)						
Psoriasis	17/2762 (0.6)	38/11,048 (0.3)	1.80 (1.01–3.19)	0.045*	1.81 (1.09–3.00)	0.021*
Control	2745/2762 (99.4)	11,010/11,048 (99.7)	1		1	
Age ≥ 45 years old (n = 9375)						
Psoriasis	24/1875 (1.3)	82/7500 (1.1)	1.17 (0.74–1.85)	0.495	1.17 (0.80–1.70)	0.421
Control	1851/1875 (98.7)	7418/7500 (98.9)	1		1	
Male (n = 12,010)						
Psoriasis	21/2402 (0.9)	67/9608 (0.7)	1.26 (0.77–2.05)	0.364	1.27 (0.84–1.92)	0.251
Control	2381/2402 (99.1)	9541/9608 (99.3)	1		1	
Female (n = 11,175)						
Psoriasis	20/2235 (0.9)	53/8940 (0.6)	1.51 (0.90–2.54)	0.115	1.49 (0.96–2.31)	0.077
Control	2215/2235 (99.1)	8887/8940 (99.4)	1		1	
Low income group (n = 11,795)						
Psoriasis	21/2359 (0.9)	59/9436 (0.6)	1.43 (0.87–2.35)	0.163	1.39 (0.91–2.11)	0.128
Control	2338/2359 (99.1)	9377/9436 (99.4)	1		1	
High income group (n = 11,390)						
Psoriasis	20/2278 (0.9)	61/9112 (0.7)	1.31 (0.79–2.18)	0.291	1.34 (0.87–2.06)	0.178
Control	2258/2278 (99.1)	9051/9112 (99.3)	1		1	
Urban resident (n = 10,610)						
Psoriasis	19/2122 (0.9)	49/8488 (0.6)	1.56 (0.91–2.65)	0.103	1.55 (0.98–2.45)	0.059
Control	2103/2122 (99.1)	8439/8488 (99.4)	1		1	
Rural resident (n = 12,575)						
Psoriasis	22/2515 (0.9)	71/10,060 (0.7)	1.24 (0.77–2.01)	0.377	1.24 (0.84–1.85)	0.282
Control	2493/2515 (99.1)	9989/10,060 (99.3)	1		1	
CCI scores = 0 (n = 17,556)						
Psoriasis	21/3320 (0.6)	77/14,236 (0.5)	1.17 (0.72–1.90)	0.524	1.20 (0.81–1.77)	0.366
Control	3299/3320 (99.4)	14,159/14,236 (99.5)	1		1	
CCI scores = 1 (n = 2787)						
Psoriasis	9/645 (1.4)	20/2142 (0.9)	1.50 (0.68–3.31)	0.314	1.63 (0.79–3.36)	0.183
Control	636/645 (98.6)	2122/2142 (99.1)	1		1	
CCI scores ≥ 2 (n = 2842)						
Psoriasis	11/672 (1.6)	23/2170 (1.1)	1.55 (0.75–3.20)	0.233	1.58 (0.82–3.03)	0.173
Control	661/672 (98.4)	2147/2170 (98.9)	1		1	

Abbreviations: CCI, Charlson Comorbidity Index; CD, Crohn's disease.
* Significance at $p < 0.05$.
† Adjusted for age, sex, income, region of residence, and CCI scores.

Table S3 Crude and overlap propensity score weighted odd ratios of psoriasis for UC

Characteristics	N of UC	N of Control	Odd ratios for UC (95% confidence interval)			
	(exposure/total, %)	(exposure/total, %)	Crude	p value	Overlap weighted model †	p value
Total participants (n = 31,645)						
Psoriasis	100/6329 (1.6)	229/25,316 (0.9)	1.76 (1.39–2.23)	<0.001*	1.77 (1.44–2.18)	<0.001*
Control	6229/6329 (98.4)	25,087/25,316 (99.1)	1		1	
Age < 45 years old (n = 13,380)						
Psoriasis	21/2676 (0.8)	56/10,704 (0.5)	1.50 (0.91–2.49)	0.112	1.52 (0.99–2.33)	0.057
Control	2655/2676 (99.2)	10,648/10,704 (99.5)	1		1	
Age ≥ 45 years old (n = 18,265)						
Psoriasis	79/3653 (2.2)	173/14,612 (1.2)	1.84 (1.41–2.41)	<0.001*	1.86 (1.47–2.36)	<0.001*
Control	3574/3653 (97.8)	14,439/14,612 (98.8)	1		1	
Male (n = 16,725)						
Psoriasis	68/3345 (2.0)	136/13,380 (1.0)	2.02 (1.51–2.71)	<0.001*	2.03 (1.56–2.65)	<0.001*
Control	3277/3345 (98.0)	13,244/13,380 (99.0)	1		1	
Female (n = 14,920)						
Psoriasis	32/2984 (1.1)	93/11,936 (0.8)	1.38 (0.92–2.07)	0.117	1.40 (1.00–1.97)	0.051
Control	2952/2984 (98.9)	11,843/11,936 (99.2)	1		1	
Low income group (n = 15,100)						
Psoriasis	49/3020 (1.6)	103/12,080 (0.9)	1.92 (1.36–2.70)	<0.001*	1.94 (1.43–2.63)	<0.001*
Control	2971/3020 (98.4)	11,977/12,080 (99.1)	1		1	
High income group (n = 16,545)						
Psoriasis	51/3309 (1.5)	126/13,236 (1.0)	1.63 (1.18–2.26)	0.003*	1.64 (1.24–2.18)	<0.001*
Control	3258/3309 (98.5)	13,110/13,236 (99.0)	1		1	
Urban resident (n = 13,800)						
Psoriasis	42/2760 (1.5)	90/11,040 (0.8)	1.88 (1.30–2.72)	<0.001*	1.88 (1.36–2.60)	<0.001*
Control	2718/2760 (98.5)	10,950/11,040 (99.2)	1		1	
Rural resident (n = 17,845)						
Psoriasis	58/3569 (1.6)	139/14,276 (1.0)	1.68 (1.24–2.29)	<0.001*	1.71 (1.30–2.23)	<0.001*
Control	3511/3569 (98.4)	14,137/14,276 (99.0)	1		1	
CCI scores = 0 (n = 22,768)						
Psoriasis	64/4252 (1.5)	147/18,516 (0.8)	1.91 (1.42–2.57)	<0.001*	1.94 (1.50–2.51)	<0.001*
Control	4188/4252 (98.5)	18,369/18,516 (99.2)	1		1	
CCI scores = 1 (n = 4011)						
Psoriasis	11/947 (1.2)	37/3064 (1.2)	0.96 (0.49–1.89)	0.91	1.04 (0.58–1.86)	0.896
Control	936/947 (98.8)	3027/3064 (98.8)	1		1	
CCI scores ≥ 2 (n = 4866)						
Psoriasis	25/1130 (2.2)	45/3736 (1.2)	1.86 (1.13–3.04)	0.014*	1.92 (1.22–3.04)	0.005*
Control	1105/1130 (97.8)	3691/3736 (98.8)	1		1	

Abbreviations: CCI, Charlson Comorbidity Index; UC, Ulcerative colitis.

* Significance at $p < 0.05$.

† Adjusted for age, sex, income, region of residence, and CCI scores.
